# Long-Acting Beta Agonists Enhance Allergic Airway Disease

**DOI:** 10.1371/journal.pone.0142212

**Published:** 2015-11-25

**Authors:** John M. Knight, Garbo Mak, Joanne Shaw, Paul Porter, Catherine McDermott, Luz Roberts, Ran You, Xiaoyi Yuan, Valentine O. Millien, Yuping Qian, Li-Zhen Song, Vincent Frazier, Choel Kim, Jeong Joo Kim, Richard A. Bond, Joshua D. Milner, Yuan Zhang, Pijus K. Mandal, Amber Luong, Farrah Kheradmand, John S. McMurray, David B. Corry

**Affiliations:** 1 Departments of Pathology & Immunology and Medicine, Baylor College of Medicine, Houston, Texas, United States of America; 2 Department of Medicine, Baylor College of Medicine, Houston, Texas, United States of America; 3 Department of Otorhinolaryngolgy – Head and Neck Surgery, University of Texas Medical School at Houston, Houston, Texas, United States of America; 4 Department of Medicine and the Translational Biology and Molecular Medicine Program, Baylor College of Medicine, Houston, Texas, United States of America; 5 Departments of Pharmacology, and Biochemistry & Molecular Biology, Baylor College of Medicine, Houston, Texas, United States of America; 6 Department of Biochemistry & Molecular Biology, Baylor College of Medicine, Houston, Texas, United States of America; 7 Department of Pharmacological and Pharmaceutical Sciences, University of Houston College of Pharmacy, Houston, Texas, United States of America; 8 Laboratory of Allergic Diseases, National Institutes of Allergic and Infectious Disease, National Institutes of Health, Bethesda, Maryland, United States of America; 9 Department of Experimental Therapeutics, The University of Texas MD Anderson Cancer Center, Houston, Texas, United States of America; 10 Department of Experimental Therapeutics, The University of Texas MD Anderson Cancer Center and the Center for Immunology and Autoimmune Diseases, The Brown Foundation Institute of Molecular Medicine for the Prevention of Human Diseases, University of Texas Medical School at Houston, Houston, Texas, United States of America; 11 Departments of Medicine and Pathology & Immunology, Translational Biology and Molecular Medicine Program, and the Biology of Inflammation Center, Baylor College of Medicine and the Michael E. DeBakey VA Center for Translational Research on Inflammatory Diseases, Houston, Texas, United States of America; French National Centre for Scientific Research, FRANCE

## Abstract

Asthma is one of the most common of medical illnesses and is treated in part by drugs that activate the beta-2-adrenoceptor (β_2_-AR) to dilate obstructed airways. Such drugs include long acting beta agonists (LABAs) that are paradoxically linked to excess asthma-related mortality. Here we show that LABAs such as salmeterol and structurally related β_2_-AR drugs such as formoterol and carvedilol, but not short-acting agonists (SABAs) such as albuterol, promote exaggerated asthma-like allergic airway disease and enhanced airway constriction in mice. We demonstrate that salmeterol aberrantly promotes activation of the allergic disease-related transcription factor signal transducer and activator of transcription 6 (STAT6) in multiple mouse and human cells. A novel inhibitor of STAT6, PM-242H, inhibited initiation of allergic disease induced by airway fungal challenge, reversed established allergic airway disease in mice, and blocked salmeterol-dependent enhanced allergic airway disease. Thus, structurally related β_2_-AR ligands aberrantly activate STAT6 and promote allergic airway disease. This untoward pharmacological property likely explains adverse outcomes observed with LABAs, which may be overcome by agents that antagonize STAT6.

## Introduction

The obstructive airway diseases, including asthma, the smoking-related chronic obstructive pulmonary diseases (COPD) chronic bronchitis and emphysema, and many other disorders are all marked in affected individuals by the presence of abnormal airways having fixed or reversible obstruction that increases the work of breathing. This abnormal physiological state is perceived as shortness of breath (dyspnea), and when severe can result in hypoxemia and death due to asphyxiation. Despite having markedly different environmental and immunological causes, asthma and COPD are both treated by agents that broadly inhibit inflammation (glucocorticosteroids) and drugs that relax airway smooth muscle to promote bronchodilation [1, 2].

Diverse bronchodilating agents have been used therapeutically in obstructive airway diseases for more than a century, beginning with the introduction of crude adrenal gland extracts from animals and soon thereafter with purified injectable preparations of the hormone epinephrine (adrenaline) that proved to be salutary in asthma. While unquestionably an effective bronchodilator, epinephrine, the only known natural ligand for the beta-2 adrenoceptor (β_2_-AR), has numerous undesirable side effects that necessitated the creation of chemical congeners having enhanced specificity for the β_2_-AR and greater stability. Each subsequent generation of β_2_-AR agonists has been linked to excess asthma-related mortality. The first generation of these agents included isoproterenol and fenoterol, which were soon linked to excess asthma-related mortality approximately 50 years ago in Europe, Australia and New Zealand [3, 4]. Whether due to cardiovascular toxicity, masking of worsening asthma or another cause, reducing the use of these drugs ultimately solved this problem [5].

Currently the most widely used beta agonist bronchodilators for use in asthma include second-generation short acting agents (SABAs) such as albuterol and long-acting agents (LABAs) with up to 12 hours of activity such as salmeterol and formoterol. Ultra-long-acting agents, having activity as long as 24 hours such as indacaterol, are now approved for use in Europe and the United States [6]. While highly effective in providing immediate relief of bronchoconstriction, emerging studies suggest that the converse strategy, β_2_-AR blockade (beta blockade) with certain beta-blockers such as nadolol, may be more effective for chronic asthma management [7–10]. The structures of diverse β_2_-AR ligands are shown in S1 Fig.

LABAs such as salmeterol and formoterol have become preferred inhaled asthma drugs because they improve disease control while requiring less frequent dosing relative to the older SABAs [6]. However, like first generation beta agonists, salmeterol has been linked in a small subset of patients to loss of disease control and excess asthma-related mortality [11]. The mechanism for this toxicity is unknown, but the improved specificity of LABAs for the β_2_-AR suggests that the cardiovascular toxicity seen in earlier generation agents is an unlikely cause. In addition to the canonical G protein coupled adenylyl cyclase signaling pathway that is induced by SABAs, LABAs also signal through the β_2_-AR to activate a biased pathway involving the adapter protein beta arrestin 2 (βarr2) and which activates extracellular-signal regulated kinases 1/2 (ERK 1/2) [12, 13]. While it is clear that adenylyl cyclase and cyclic GMP mediate bronchodilation, the long-term biological consequences of βarr2-dependent signaling through the β_2_-AR are unknown. Nonetheless, the United States Food and Drug Administration now mandates that LABAs be co-administered with an inhaled glucocorticosteroid as such combination therapy appears to ameliorate morbidity and mortality related to salmeterol, but not formoterol, monotherapy [14–16]. Because inhaled glucorticosteroids are immune suppressants, the partial palliative effect of these agents suggests that LABA-related toxicity may have an inflammatory basis.

Over the last two decades, the immunological nature of allergic asthma has gradually become clear through the study of experimental systems. Central to the generation of airway obstruction in the context of allergic inflammation are T cells, especially T helper type 2 (T_H_2) cells that secrete interleukin 4 (IL-4), IL-5 and IL-13 and Th17 cells that produce the cytokines IL-17A and IL-17F. Th17 cells partner with T_H_2 cells through a complex mechanism to promote IL-13-dependent airway hyperresponsiveness, the exaggerated tendency of the asthmatic airway to constrict in response to diverse stimuli [17–20]. The airway epithelium, through secreted cytokines that include IL-25 and thymic stromal lymphopoietin (TSLP) and endogenous proteinases such as matrix metalloproteinase 7 (MMP7), and novel proteinase-activated inflammatory pathways that include fibrinogen and Toll like receptor 4 (TLR4), further promote airway hyperresponsiveness and allergic inflammation [21–25]. Despite such complexity, the diverse signal transduction pathways that are relevant to asthma appear to converge on the singularly important pathway that includes the ligands IL-4 and IL-13 [19, 20, 26]; the receptor signaling chain alpha chain of the IL-4 receptor (IL-4Rα) [19, 27]; and the transcription factor signal transducer and activator of transcription 6 (STAT6)[28, 29]. Underscoring the importance of this pathway are monoclonal antibodies against IL-13 and IL-4Rα that have shown considerable promise in early phase asthma clinical trials [30, 31].

In this study, we explored the mechanism of LABA-dependent toxicity in the context of allergic airway disease using a combination of in vivo models and mouse and human primary and transformed cell lines. Our findings reveal a novel immune mechanism by which LABAs and structurally related β_2_-AR ligands that include beta-blockers such as carvedilol, promote exaggerated allergic inflammation by aberrantly activating STAT6. We show using a specific antagonist of STAT6 activation that such toxicity can be abrogated, revealing a new approach to the management of asthma and related allergic disorders.

## Materials and Methods

All studies were conducted at Baylor College of Medicine, MD Anderson Cancer Center, and the National Institutes of Health in accordance with all applicable government and institutional guidelines. Allergic airway disease was induced in mice with a clinical isolate of the fungus *Aspergillus niger* according to our previously established procedures [26, 32]. Synthesis of PM-242H was based on a previously published method for the synthesis of antagonists of STAT3 [33].

### Human Subjects Research

Peripheral blood mononuclear cells (PBMC) isolated from healthy donors were used as sources of human T cells that were activated and stained for intracellular STAT6 expression as previously described [34] and using reagents as noted in the text. Use of human PBMC was approved by the Institutional Review Board at the National Institutes of Health and patients gave written consent. Consent is documented in the patient's NIH electronic medical research record system, CRIS, by the consenting protocol investigator, and the actual written and witnessed consents are scanned into the NIAID electronic medical research system, CRIMSON.

### Mice

Female mice between the ages of 4 and 8 weeks (18–25 grams) were used. All studies were conduced in compliance with the Baylor College of Medicine Institutional Animal Care and Use Committee (BCM IACUC) regulations as approved through protocol AN-1819. β-arrestin 2 knockout (βarr2^–/–^; 8 generations backcrossed to the C57BL/6 background) and β_2_ Adrenergic Receptor knockout (β_2_AR^–/–^; eight generations backcrossed to the FVB background) mice were generated as previously described [35, 36]. Balb/c, C57BL/6, and FVB wild type mice were purchased from Jackson Laboratories (Bar Harbor, ME) and used either alone or as wild type controls for gene-deficient mice. All mice were housed under SPF conditions in the transgenic mouse facility at Baylor College of Medicine, given standard rodent chow and sterile water ad libitum, provided 12h/12h light/dark cycles, housed on standard corn cob sterilized bedding and given daily welfare checks by husbandry staff.

### Drugs and Synthetic Reagents

Salmeterol (SX; S5068), albuterol (PHR1053), carvedilol (C3993), and nadolol (N1892) were purchased from Sigma-Aldrich (St. Louis, MO). 1,2-dilauroyl-sn-glycero-3-phosphocholine (DLPC; 770335) was purchased from Avanti Polar Lipids (Alabaster, AL) and used as a vehicle at a 1:5 (drug:DLPC) ratio. In brief, drug and DLPC were solubilized in *tert*-butanol, frozen, lyophilized, and then placed in fine suspension by the addition of sterile, endotoxin-free phosphate buffered saline (PBS) and sonicated at 60 Hz for 30 seconds prior to use in vitro studies and administration to mice.

### Synthesis of PM-242H

Synthesis of the STAT6 antagonist PM-242H was based on previously published methods [37, 38] with the strategy summarized in S2 Fig.

Reagents and conditions: i) 4-aminobiphenyl, EDC, CH_2_Cl_2_, rt, 12h, 91%; ii) Ph_3_Bi, Cu(OAc)_2_, TEA, CH_2_Cl_2_, rt, 48h, 77%; iii) a) TFA, b) Fmoc-Tle-OH, HBTU, DIPEA, rt, 12h, 82%; iv) 20% piperidine/DMF 30min. 61%; v) **6**, NMP, NMM, DMAP(cat.), 2h, 71%.

#### Synthesis of Boc-prolyl-4-amidobiphenyl 2

A solution of Boc-Proline (**1**, 2.0 g, 9.3 mmol), 4-aminobiphenyl (1.6 g, 9.3 mmol) and EDC (2.1 g, 11.2 mmol) in 60 mL of dry CH_2_Cl_2_ was stirred overnight. It was then transferred to a separatory funnel with an additional 20 mL of CH_2_Cl_2_ and washed with 5% HCl (2 × 30mL) followed by 10% NaHCO_3_ (2 × 30mL) and brine (1 × 20mL). The organic layer was dried (MgSO_4_) and concentrated under reduced pressure. Purification by silica gel column chromatography eluting with 15% EtOAc-hexane afforded the title products as a white solid (3.1 g, 91% yield). Calcd (M+H): 367.2022; Found (M+H): 367.2351. ^1^H NMR (CDCl_3_, 600MHz) δ:9.6 (s, 1H), 7.51–7.63(m, 6H), 7.44 (m, 2H), 7.34 (m, 1H), 4.54 (m, 1H), 3.32–3.68(m, 2H), 1.84–2.07(m, 4H), 1.54(s, 9H).^13^C NMR(CDCl_3_, 150MHz) δ: 128.9, 127.5, 127.0, 126.8, 119.9, 80.9, 47.3, 28.4.

#### Synthesis of N-phenyl Boc-prolyl-4-amidobiphenyl, 3

To a stirred solution of **2** (2.0 g, 5.4 mmol) in dry CH_2_Cl_2_ (50 mL) was added triphenylbismuth (3.6 g, 8.2 mmol), Cu(OAc)_2_ (1.6g, 8.2 mmol) and dry triethylamine (1.2 mL, 8.2 mmol). The reaction was monitored by HPLC. After completion of the reaction, the solvent was evaporated *in vacuo* and the residue was diluted with ether (150 mL) and filtered through celite. The organic layer was washed with 5% HCl (2 × 30 mL) followed by brine and was dried over MgSO_4_. Concentration under reduced pressure followed by purification by silica gel chromatography using 10% EtOAc-hexane afforded **3** as a white solid (1.9 g, 77% yield). Calcd (M+H): 443.2335; Found (M+H): 443.2349 ^1^H NMR (CDCl_3_, 600MHz) δ: 7.2–7.63 (m, 14H), 4.38 (m, 1H), 4.25 (m, 1H isomer), 3.48–3.6 (m, 2H), 3.4 (m, 1H), 3.32(m, 1H isomer), 1.8–2.1(m, 2H), 1.65–1.75 (m, 2H), 1.46 (s, 9H), 1.4 (s, 9H isomer). ^13^C NMR (CDCl_3_, 150MHz) δ: 173.2, 172.8, 154.4, 153.8, 129.9, 129.3, 128.8, 128.4, 127.5, 127.1, 126.4, 125.9, 79.9, 79.3, 58.00, 57.8, 47.2, 31.9, 30.4, 28.8, 28.6, 28.4, 24.3, 23.4.

#### Synthesis of Fmoc-tert-butylglycyl-N-phenyl-prolyl-4-amidobiphenyl, 4

A solution of **3** (1.00 g, 2.25 mmol) in 5 mL of neat trifluoroacetic acid (TFA) was stirred for 1h. Excess TFA was removed under vacuum. The residue was then treated with Fmoc-Tle-OH (0.8 g, 2.25 mmol), HBTU (0.85 g, 2.25 mmol), DIPEA (1.2 mL, 6.7 mmol) in 50 mL of dry CH_2_Cl_2_ overnight. The organic layer was diluted with an additional 50 mL of CH_2_Cl_2_ and washed with 5% HCl (3 × 30 mL) followed by 10% NaHCO_3_ (1 × 30 mL) and brine. After drying (MgSO_4_) and concentration under vacuum, the crude product was purified by silica gel chromatography using 35% EtOAc-hexane to give the desired material as white foam. Yield: 1.25 g 82%. Calcd (M+H): 678.3332; Found (M+H): 678.3438. ^1^H NMR (CDCl_3_, 600MHz) δ: 7.78 (m, 2H), 7.63–7.71 (m, 3H), 7.24–7.62 (m, 17H), 6.5 (d, J = 10.5Hz, 1H), 4.67 (m, 1H), 4.6 (m, 1H, isomer), 4.5 (d, J = 10.5Hz, 1H),4.42 (m, 1H), 4.3 (m, 1H), 4.23 (m, 1H), 4.0 (m, 1H), 3.84 (m, 1H), 2.1–2.24 (m, 3H), 1.94 (m, 1H),1.17 (s, 9H). ^13^C NMR (CDCl_3_, 150 MHz) δ:172.5, 171.3, 159.1, 158.8, 156.9, 143.8, 142.1, 141.7, 141.3, 140.8, 140.4, 139.8, 130.1, 129.3, 128.9, 128.7, 127.9, 127.8, 127.7, 127.4, 127.1, 127.0, 126.6, 126.5, 125.4, 125.3, 120.0, 119.9, 115.8, 113.9, 67.5, 59.5, 59.3, 49.3, 47.1, 35.9, 29.7, 26.4, 25.7, 25.3.

#### Synthesis of tert-butylglycyl-N-phenyl-prolyl-4-amidobiphenyl, 5

Compound **4**, (0.5 g, 0.74 mmol) was treated with 4.0 mL of 20% piperidine in DMF for 30 min. The reaction mixture was concentrated under vacuum. The residue was purified by to RP-HPLC (2.5 × 25 cm Phenomonex Luna C18 column eluting with a linear gradient of MeCN in H_2_O) and the pure fractions then collected and lyophilized to get 0.210 g (61% yield) of desired material (**5**) as a white powder. Calcd (M+H): 456.2651; Found (M+H): 456.2708.

#### Synthesis of PM-242H

To a stirred solution of **5** (0.05g, 0.1mmol) and the active ester (**6**)^1^ (0.075g, 0.1mmol) in 3mL of dry NMP, 40 μL of N-methylmorpholine and 4-DMAP (0.002g, 0.02mmol) was added. The reaction was then monitored by HPLC. After completion, the desire product then purified from the crude by RP-HPLC (2.5 × 25 cm Phenomonex Luna C18 column eluting with a linear gradient of MeCN in H_2_O). The combined pure fractions were then lyophilized to get pure **PM-242H** as a white powder (73 mg, 71%). Calcd (M +H): 944.4063; Found (M+H): 944.4217. ^1^H NMR (CDCl_3_, 600MHz) δ: 7.5–7.56 (m, 4H), 7.4–7.5 (m, 7H), 7.3–7.38 (m, 3H), 7.2–7.3 (m, 5H), 6.82 (d, J = 10.5 Hz, 1H), 6.44 (d, J = 16.0Hz, 1H), 5.66 (m, 2H), 5.57 (m, 2H), 4.8 (d, J = 10.5Hz, 1H), 3.9 (m, 1H), 3.75 (m, 1H), 1.93–2.12 (m, 3H), 1.8 (m, 1H), 1.14 (s, 18H), 1.07 (s, 9H). ^13^C NMR (CDCl_3_, 150MHz) δ:176.6, 172.0, 170.3, 165.4, 140.1, 137.6, 130.0, 129.1, 128.9, 128.8, 128.5, 127.8, 127.7, 127.1, 126.9, 126.5, 122.5, 82.5, 82.4, 59.0, 57.4, 49.1, 38.7, 36.3, 29.8, 26.7, 26.6.

### Infectious Allergic Airway Disease Model

Mice were challenged intranasally with a clinical isolate of 4 × 10^5^ *A*. *niger* conidia every two days for a total of 8 challenges and treated with 50 μg of the indicated β-agonist, liposome vehicle (DLPC) and/or PM-242H (5 or 50 μg) intranasally as indicated and the allergic airway disease phenotype was determined according to our previously described methods [32].

### Allergic Airway Disease Analysis

Allergic airway disease was assessed as previously described [32]. Changes in respiratory system resistance (R_RS_) in response to intravenous acetylcholine challenge, bronchoalveolar lavage (BAL) fluid differential counts, and analysis of lung IL-4, IL-17A and IFN-γ producing cells by enzyme linked immunocell spot assay (ELISpot) were performed as previously described [39, 40].

### Ovalbumin restimulation

Splenocytes from the indicated challenge groups of naïve and sensitized mice were assessed for antigen-specific recall cytokine responses by ELISpot. In brief, spleens were removed post mortem, de-aggregated by pressing through 40μm nylon mesh and the red blood cells were removed from resulting cell suspension by hypotonic lysis. The splenocytes were then washed twice and cultured in flat-bottom wells of 96-well microtiter plates that were pre-coated with capture antibodies to IL-4, interferon gamma (IFN-γ) and IL-17A. Splenocytes were added in duplicate cultures of 0.5 × 10^6^ cells/well that were then diluted serially two-fold to 0.015 × 10^6^ cells/well. Cells were cultured in the presence of media or whole ovalbumin (1 mg/mL) overnight and plates were developed as previously described [41].

### Histology

Lungs were perfused of blood by cannulating the pulmonary artery and injecting ice cold PBS until lavage returning via the left atrium was clear. Lungs were then inflated via the trachea with 10% formalin at 25 cm water pressure and the tracheas were tied off prior to removal of the cardiopulmonary unit en bloc submerging in 10% formalin overnight. Fixed lungs were then divided into individual lobes that were then halved and embedded in paraffin. Lung sections were cut at 5 microns and stained with the periodic acid-Schiff (PAS) kit (395B; Sigma-Aldrich, St. Louis, MO).

### Cell Culture

Mycoplasma-free A549 (CCL-185) cells, derived from a human lung adenocarcinoma of epithelial origin, were acquired from American Type Culture Collection (Manassas, VA). Cells were cultured in 50% DMEM, 50% F-12 complete media until confluent and switched to culture media containing 2% FBS for at least 24h before stimulation. Long-term cultures were initiated on confluent cells by the addition of vehicle (DLPC) or select β-agonists and blockers (albuterol, salmeterol and nadolol) at a working concentration of 10 μM. Cells were stimulated with 2 ng/mL IL-13 (213-IL/CF; R&D Systems, Minneapolis, MN) for identified times and harvested for protein and mRNA. Recombinant IL-6 (10 ng/mL) and IL-2 (5 ng/mL) were also added as indicated (both from R&D Systems).

Mycoplasma-free primary human epithelial cells of the sinonasal cavity were isolated from chronic rhinosinusitis patients and plated in complete BEGEM on type I collagen coated plates as previously described [42]. Confluent cultures were maintained in the presence of vehicle (DLPC), 10 μM albuterol or 10 μM salmeterol for 4–5 days with media refreshed daily. On day 5, cells were stimulated for 24 hours with 2 ng/mL recombinant human IL-13.

### Western Blot

Cells or lungs were isolated and lysed in RIPA buffer (9806S; Cell Signaling, Danvers, MA), protein was quantified using BCA Protein Assay Reagent (23227; Thermo Fisher Scientific, Rockford, IL) and denatured in Laemmli sample buffer (161–0737; Bio-Rad, Hercules, CA) according to manufacturer’s protocol. Proteins were separated on SDS-Page gels were hand-poured using a standard electrophoresis unit (Bio-Rad, Hecules, CA) and transferred to a membrane using iBlot Gel Transfer Device (IB1001; Invitrogen, Grand Island, NY). Membranes were blocked in 2% FBS PBST and probed for c-Src, p-c-Src, pErk1/2, Erk1/2, pStat6, Stat6, pStat3, Stat3, pStat5, Stat5, Shp-1, and β-actin. Signal was detected with a ChemiDoc XRS+ system (BioRad; Hercules, CA).

### qPCR

RNA was purified from cell culture or select lobes of lung identified mice and RNA using the RNeasy Mini Kit (74104; Qiagen, Valencia, CA) or Trizol (15596–026; Invitrogen, Grand Island, NY) and cDNA made with the TaqMan Reverse Transcription kit (N8080234; Applied Biosystems, Foster City, CA). Probes were acquired from Applied Biosystems (Foster City, CA) for 18s, CC26 and Muc5AC and used to quantify relative expression with TaqMan Fast Universal PCR Master Mix (435189, Applied Biosystems, Foster City, CA) on a the 7500 Real-Time PCR System (Applied Biosystems, Foster City, CA).

### Fungal Burden

Fungal burden of fungal challenged mice was assessed by serial dilution of lung homogenates (1/10 volume) on Sabouraud agar plates (84088; Sigma Aldrich, St. Louis, MO) in the presence of 100 mg/mL chloramphenicol (C0378; Sigma Aldrich, St. Louis, MO). Plates were incubated overnight at 37°C, assessed for total number of colonies and fungal colony forming units/lung was determined by dilution.

### Statistical and Data Analysis

Data are presented as means ± standard error of means (SEM). For paired, normally distributed (log-transformed respiratory system resistance (R_RS_)) data, Student’s T test was used to determine significance (P < 0.05). Otherwise, group comparisons of R_RS_ data were made using ANOVA with Bonferroni’s correction. All other data were compared using either the Mann-Whitney (2 groups) or Kruskal-Wallis (> 2 groups) tests. Sample sizes for animal experiments were determined based on prior studies in which n = 4 or 5 was found to be sufficient to achieve significance with regard to R_RS_ values in moderately polarized treatment groups. Mice were assigned to their treatment groups randomly. Data variation was similar between compared experimental groups. Airway physiology data were collected from randomly selected, de-identified mice and all subsequent data were analyzed from coded, de-identified samples or data sets to minimize subjectivity and bias. All data and datasets will be made available to any investigator upon request.

## Results

### Structurally related LABAs induce exaggerated allergic airway disease

To understand the molecular basis of LABA-related toxicity, we first compared the structures of commonly used LABAs and SABAs as well as β_2_-AR antagonists (beta blockers; S1 Fig). In general, the structure of SABAs was closely related to the parent compound epinephrine. For example, in isoproterenol and albuterol slightly larger isopropyl and *tertiary* butyl branched alkyl groups replace the methyl substituent on the side chain nitrogen atom. In contrast, the structures of LABAs differ significantly from epinephrine in having substantially larger aliphatic tails of varying length that extend from the side chain nitrogen and terminate in a second aromatic group. Some beta-blockers such as carvedilol also possess an alkyl-aryl extension on the corresponding nitrogen atom similar to the LABAs.

**Fig 1:**
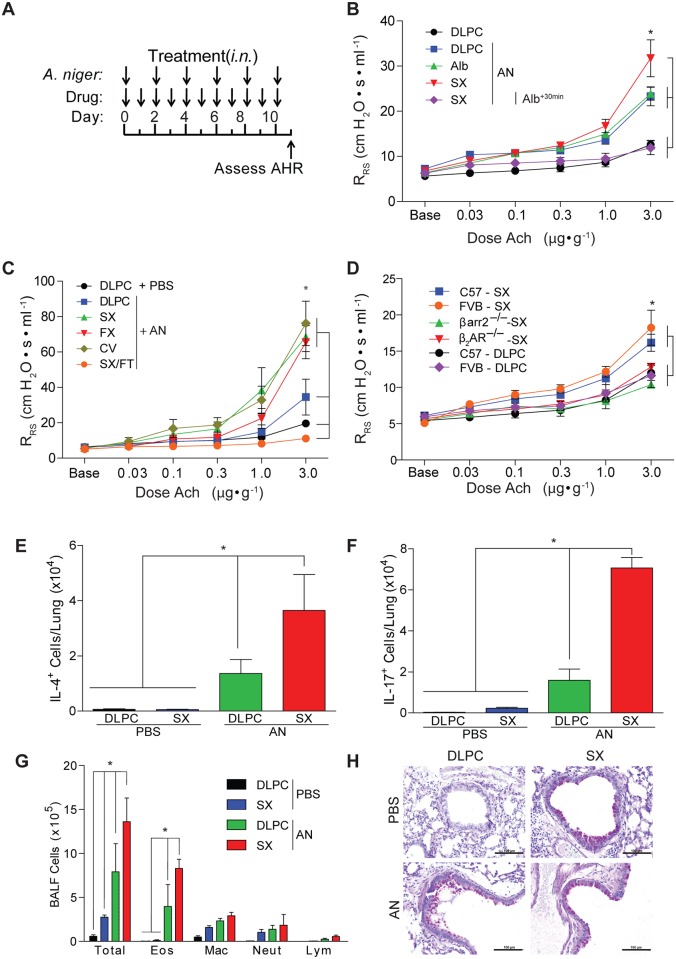
Long acting beta agonists promote allergic airway disease. (**a**) Wild type mice were challenged every other day intranasally (i.n.) over 10 days with the spores of *A*. *niger* (AN) and every day as indicated with the drugs salmeterol (SX), formoterol (FX), carvedilol (CV), albuterol (Alb) or salmeterol or the combination of salmeterol and fluticasone (SX/FT) and compared to mice challenged with vehicles (PBS and dilauroylphosphatidylcholine (DLPC) liposomes) and (**b, c**) the effect on airway hyperresponsiveness (AHR) was determined (all data from C57BL/6 mice). *: P < 0.05 determined by ANOVA. (**d**) Genotype matched wild type and β_2_-AR- and ßarr2-deficient mice were challenged with salmeterol or DLPC alone i.n. and assessed for airway hyperresponsiveness. *: P < 0.05 determined by ANOVA. The effect of salmeterol on lung (**e**) IL-4- and (**f**) IL-17A-secreting cells, (**g**) total bronchoalveolar lavage fluid (BALF) inflammatory cells (eos: eosinophils; mac: macrophages; lym: lymphocytes; neut: neutrophils) and (**h**) airway goblet cell metaplasia was determined after *A*. *niger* challenge i.n. *: P < 0.05 determined by Mann Whitney test. Data are from one of 3 or more independent and comparable biological experiments with n = 4 mice/treatment group.

To explore the biological activities of β_2_-AR ligands with distinct structures, we developed a mouse model of LABA-dependent lung toxicity to test the hypothesis that LABAs exacerbate established allergic airway disease. Prior experimental studies suggested that salmeterol monotherapy leads to loss of disease control in mice challenged with the allergen ovalbumin and that such effects are reversed by the addition of a corticosteroid [43]. To determine if enhanced allergic disease is a general attribute of LABAs, we assessed the effects of two widely prescribed LABAs, salmeterol and formoterol, on the expression of allergic airway disease using a fungal infectious model (Fig 1a) [32]. Compared to mice challenged with phosphate buffered saline and dilauroylphosphatidylcholine (PBS and DLPC; vehicles for delivery of fungal spores and β_2_-AR ligands, respectively) alone, mice challenged with DLPC and the spores of *Aspergillus niger* developed airway hyperresponsiveness to acetylcholine (Ach) provocation (Fig 1b). In comparison to these animals, whereas addition of the SABA albuterol had no effect on *A*. *niger*-induced airway hyperresponsiveness, confirming a prior study of mice sensitized against ovalbumin [44], mice challenged with the LABA salmeterol and *A*. *niger* developed significantly greater airway hyperresponsiveness (Fig 1b). Given the relatively long duration of the challenge protocol, we considered the possibility that salmeterol was downregulating expression of the ß_2_-AR, a process termed tachyphylaxis [45], thereby potentially suppressing the ability of endogenous adrenaline to limit expression of airway hyperresponsiveness. However, administration of albuterol 30 minutes prior to measuring airway responsiveness completely suppressed bronchoconstriction even at the highest dose of Ach given, indicating that the β_2_-AR remained fully functional (Fig 1b).

We further examined airway mechanics in fungus-challenged mice that received the structurally related LABA formoterol and beta-blocker carvedilol and found similarly enhanced airway hyperresponsiveness. In contrast, fungus-challenged mice that received the corticosteroid fluticasone and salmeterol showed complete abrogation of airway hyperresponsiveness (Fig 1c). Thus, structurally related β_2_-AR ligands, whether classified as agonist or antagonist at the canonical Gs pathway, enhance airway hyperresponsiveness in fungus-challenged mice through a unique mechanism that did not involve tachyphylaxis.

We further explored the specificity of salmeterol-induced enhanced airway hyperresponsiveness for the β_2_-AR. Wild type mice (C57BL/6 and FVB backgrounds) developed mild airway hyperresponsiveness when challenged with salmeterol alone (Fig 1d), suggesting that the disease-promoting effects of salmeterol occur independent of antigen challenge and concomitant allergic inflammation. In contrast, genetically otherwise matched mice that were deficient in the β_2_-AR (FVB background) and βarr2 (C57BL/6 background) failed to develop airway hyperresponsiveness following salmeterol challenge. Thus, salmeterol induced exaggerated airway hyperresponsiveness through a specific pathway involving the β_2_-AR and βarr2.

We also determined lung inflammatory responses in salmeterol-challenged wild type mice. Relative to mice challenged with fungal spores and vehicle, salmeterol enhanced the number of total lung cells secreting IL-4 and IL-17A, cytokines that are produced in part by T_H_2 and Th17 cells, respectively, and which contribute to the expression of both lung inflammation and airway hyperresponsiveness [17, 26, 46] (Fig 1e and 1f). Similarly, salmeterol markedly enhanced the number of airway inflammatory cells, accounted for largely by a two-fold increase in eosinophils recovered from bronchoalveolar lavage fluid in the context of fungal challenge (Fig 1g). Mucus production by goblet cells in the airway epithelium, a marker of lung remodeling that is also characteristic of asthma and the presence of IL-13 [19, 20], was induced by salmeterol even in the absence of fungus challenge (Fig 1h).

Formoterol and carvedilol also enhanced fungus-dependent IL-4 and IL-17, but not interferon gamma (IFN-γ), secretion from lung cells in comparison to fungus challenge alone and salmeterol also enhanced lung *Muc5ac* mRNA levels (S3 Fig). None of the treatments led to fungal overgrowth in the lung (S3 Fig), but the combination of salmeterol and fluticasone abrogated all inflammatory indices (S3 Fig). Thus, structurally related β_2_-AR ligands strongly enhanced the fungus-dependent expression of multiple inflammatory markers of allergic airway disease, but our studies further demonstrated intrinsic pro-asthmatic activity in salmeterol.

### Salmeterol activates STAT6

**Fig 2:**
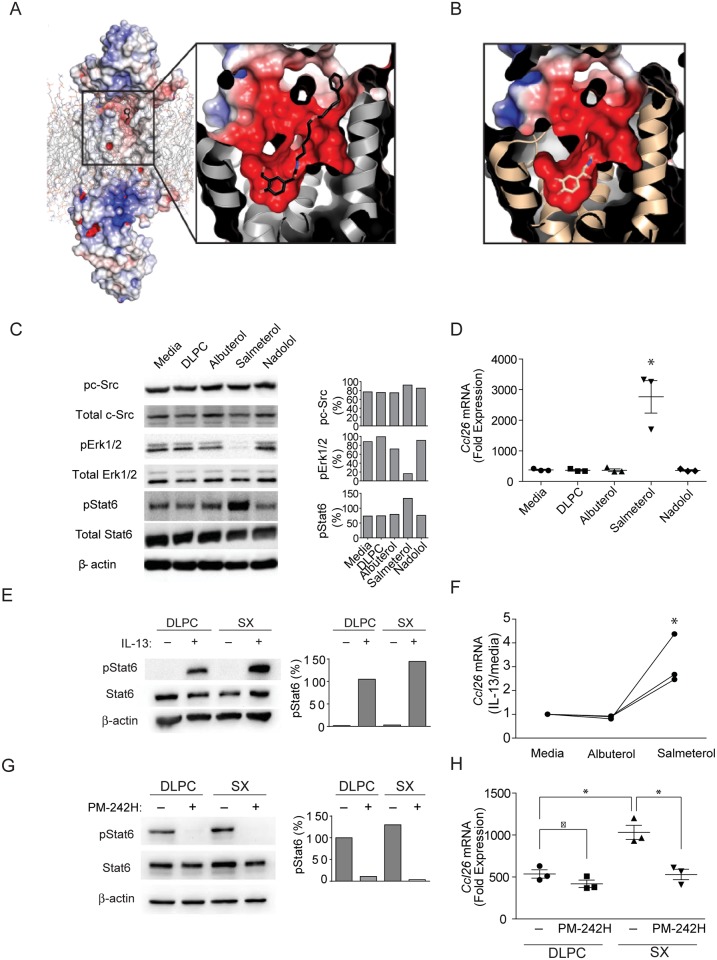
Salmeterol potentially binds aberrantly to the β_2_-AR active site and alters signaling function. (**a**) Model of the β_2_-AR bound to salmeterol. Salmeterol is docked to the β_2_-AR bound to hydroxybenzyl isoproterenol (PDB Code: 4LDE) and energy minimized using Coot [62]. The entire model is shown with electrostatic surface potential calculated with APBS [38] on the left and a zoom in view of the binding pocket is shown on the right. Salmeterol is colored by atom type, with carbon colored black, nitrogen colored blue, and oxygen colored red. (**b**) Similar rendering of the β_2_-AR binding pocket with adrenaline bound [48], but with the carbon atoms of adrenaline colored yellow. (**c-h**) A549 human airway epithelial cells were exposed to IL-13 for 5 days and then to DLPC liposome vehicle, albuterol, salmeterol, or nadolol for 5 days after which (**c**) total and phosphorylated signaling proteins were determined in relation to beta actin as indicated. (**d**) Induction of *Ccl26* mRNA as assessed by real time qPCR was determined under similar conditions in A549 cells. *: P < 0.05 determined by ANOVA (n = 3 technical replicates). (**e**) Total and phosphorylated STAT6 and (**f**) *Ccl26* mRNA were quantitated from IL-13-stimulted human bronchial epithelial cells that were exposed to salmeterol or albuterol as indicated. *: P < 0.05 determined by ANOVA (n = 3 technical replicates). The effect of the phosphopeptidomimetic PM-242H on (**g**) STAT6 phosphorylation and (**h**) *Ccl26* mRNA expression were further determined in IL-13-stimulated A549 cells. *: P < 0.05 determined by Mann Whitney test. Data are from one of 3 or more independent and comparable biological experiments.

Salmeterol, formoterol and carvedilol are physically much larger than epinephrine, raising questions regarding how these compounds bind and signal through the ß_2_-AR, especially for salmeterol, which putatively engages a non-receptor exosite that accounts for its relatively long half-life [47]. We therefore modeled the binding of salmeterol to the human β_2_-AR based on the previously established structure of the β_2_-AR bound to epinephrine [48] (Fig 2a). Assuming that salmeterol binding is homologous to that of adrenaline where these molecules resemble each other, our model indicates that salmeterol is readily accommodated in the binding site of the β_2_-AR, with no need to invoke an exosite beyond the receptor itself. Portions of salmeterol are further free to interact with regions of the β_2_-AR that are presumably not influenced by adrenaline (Fig 2b), suggesting the possibility that salmeterol can promote a unique β_2_-AR conformation and therefore function.

The β_2_-AR signals canonically through G-proteins that promote Ca^++^ flux and smooth muscle relaxation [49], but under some conditions also signals through a β-arrestin 1/2-dependent cascade to activate c-Src and Erk [50–52]. Carvedilol is an unusual β_2_-AR ligand that obligatorily biases β_2_-AR signaling through ßarr2 [53], and although classified pharmacologically as a beta blocker, it is thus also an arrestin-biasing beta agonist (ABBA). Given the ability of salmeterol to enhance allergic disease and its potential to alter β_2_-AR conformation (Fig 2a), we determined how salmeterol and other ligands influence activation of MAP kinases previously linked to β_2_-AR signaling using human A549 human lung epithelial adenocarcinoma cells, which express a functional β_2_-AR [54]. After treatment for five days with IL-13, A549 cells exposed to albuterol, salmeterol, and the beta-blocker nadolol (S1 Fig) showed no change in the expression of c-Src as compared to media or vehicle (DLPC) controls (Fig 2c). In contrast, expression of Erk1/2 was selectively suppressed in salmeterol-treated cells. We further determined the effect of these treatments on the activation of STAT6, the major transcription factor induced by IL-4 and IL-13 [55]. Salmeterol was again the only β_2_-AR ligand to cause enhanced STAT6 phosphorylation but this effect required at least four days of in vitro culture and four additional days of culture in the absence of salmeterol for STAT6 activation levels to return to baseline (S4 Fig). Salmeterol-dependent enhanced STAT6 activation was not reversed by dual therapy with salmeterol and fluticasone, in contrast to the marked anti-inflammatory effect of fluticasone in vivo (Fig 1b; S3 Fig). IL-13-induced expression of CCL26, a ligand for the T helper chemokine receptor CX3CR1 that promotes T cell survival and allergic airway disease [56, 57], was also significantly increased in salmeterol-treated A549 cells (Fig 2d). Similarly, STAT6 activation and CCL26 expression were enhanced by salmeterol in primary human bronchial epithelial cells (Fig 2e and 2f) and salmeterol further enhanced intracellular STAT6 activation after long-term exposure to human peripheral blood T cells (S5 Fig). Thus, among the diverse β_2_-AR ligands tested, salmeterol selectively suppressed MAP kinase expression while simultaneously enhancing activation of STAT6 and STAT6-dependent CCL26 secretion from airway epithelial cells.

We further sought to determine if the salmeterol-enhanced expression of CCL26 was directly linked to the enhanced activation of STAT6. For these studies we exposed IL-13 and salmeterol-stimulated airway epithelial cells to a STAT6-specific phosphopeptidomimetic inhibitor that blocks STAT6 activation through the IL-13 receptor, PM-242H. PM-242H is based on the sequence surrounding Tyr631 of the IL-4/IL-13 signaling chain IL-4 receptor alpha (IL-4Rα) that is required for STAT6 binding and function [58] and is a cell-permeable phosphopeptide mimetic prodrug that binds the SH2 domain of STAT6 (S6 Fig). PM-242H blocks STAT6 recruitment to IL-4Rα, thereby preventing subsequent activation by Janus-class kinases, dimerization, translocation to the nucleus, and transcription of IL-4 and IL-13-dependent genes. As modeled in A549 epithelial cells, PM-242H completely blocked IL-13-dependent STAT6 activation at a concentration of 10 μM. At this concentration, PM-242H further had no effect on STAT3 activation and only modest effect on the activation of STAT5 (S7 Fig). After exposure of long-term A549 cultures, PM-242H both blocked IL-13-dependent STAT6 activation and salmeterol-dependent enhanced STAT6 activation (Fig 2g). PM-242H further blocked salmeterol-dependent enhanced production of *Ccl26* mRNA (Fig 2h). Thus, salmeterol augmented CCL26 production by activating STAT6.

STAT6 is not known to be a downstream target of β_2_-adrenergic signaling, but Erk1/2 activation has previously been linked to β-arrestin/c-Src signaling in response to short-term β_2_-adrenergic and IL-13 receptor stimulation [59]. To determine if aberrant Erk1/2 suppression by salmeterol is linked to salmeterol-dependent enhancement of STAT6 activation, we cultured airway epithelial cells in the presence of IL-13 and the Erk1/2 inhibitor U0126 and found enhanced STAT6 activation similar to that induced by long-term exposure to salmeterol (S8 Fig). Thus, long-term exposure to salmeterol suppressed Erk1/2 activation, which in turn led to enhanced STAT6 activation in IL-13-stimulated epithelial cells.

### PM-242H blocks initiation of allergic airway disease and reverses established disease

**Fig 3:**
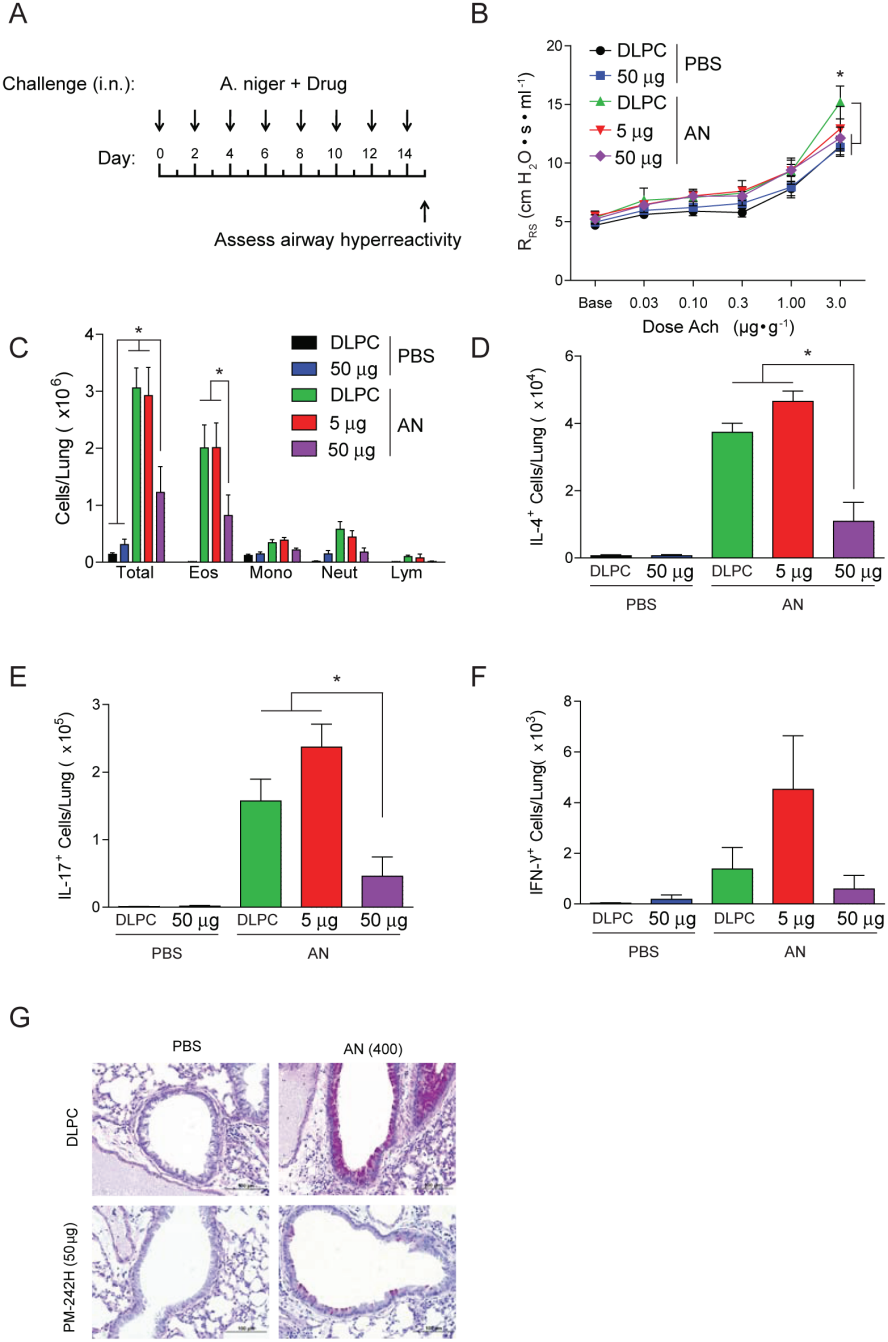
PM242H inhibits development of allergic airway disease. (**a**) Mice (C57BL/6) were treated every other day for two weeks with vehicle (DLPC) or one of two doses of PM-242H (5 or 50 μg i.n.) while also challenged every other day with 400 × 10^3^ *A*. *niger* conidia (AN) or PBS i.n. after which the allergic airway disease phenotype was assessed. (**b**) Airway resistance (*: P < 0.05 determined by ANOVA), (**c**) bronchoalveolar lavage fluid inflammatory cells, (**d**) total lung IL-4, (**e**) IL-17A, and (**f**) IFN-γ-secreting cells, and (**g**) goblet cell metaplasia from representative bronchovascular bundles are shown. *: P < 0.05 determined by Kruskal-Wallis test (n = 4 mice/treatment group). Data are from one of 4 independent and comparable biological experiments.

**Fig 4:**
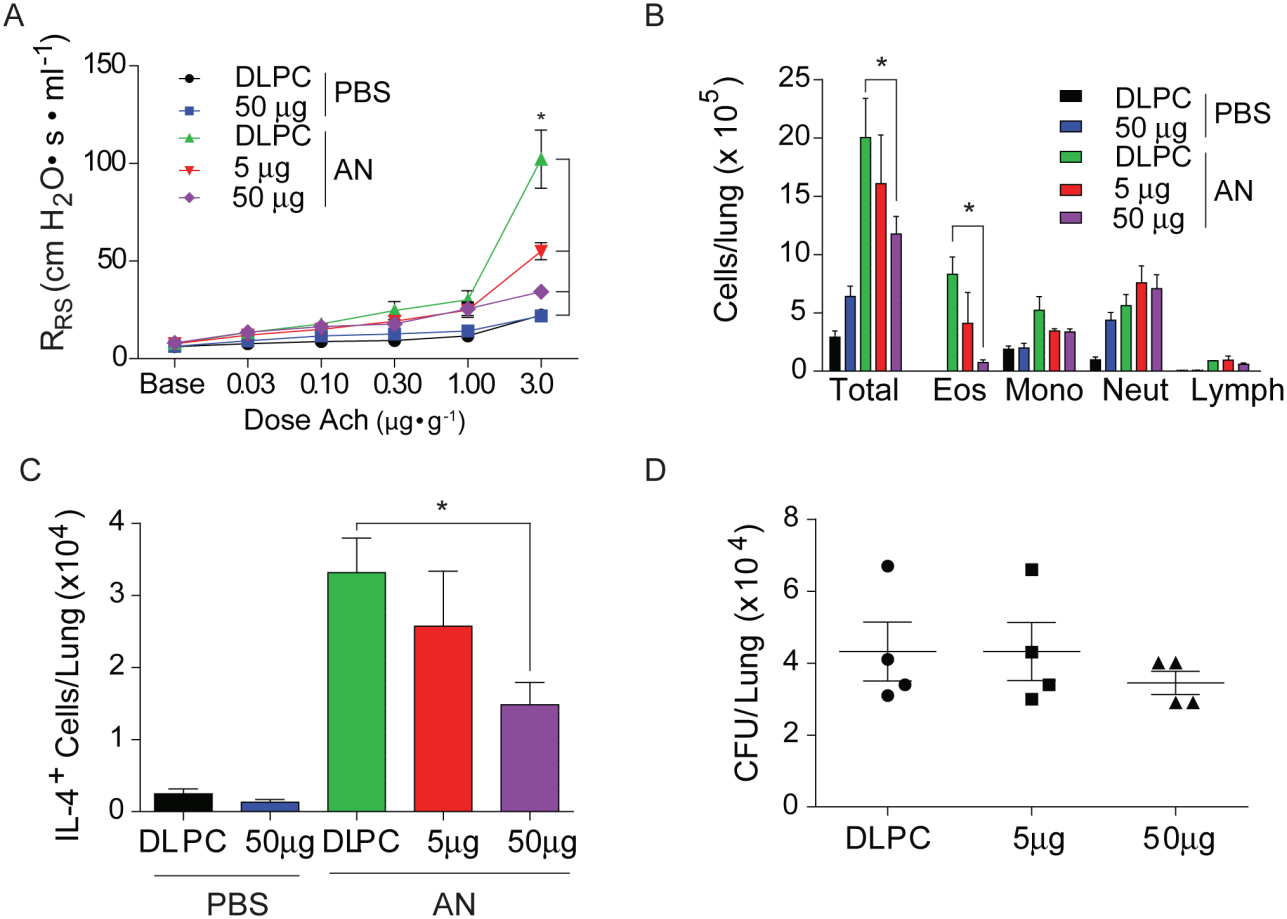
PM-242H inhibits development of allergic lung disease in Balb/c mice. Mice were treated every other day for two weeks with vehicle (DLPC) or one of two doses of PM-242H (242H; 5 mg or 50 μg i.n.) and challenged with *A*. *niger* conidia (AN) or PBS i.n. after which the allergic airway disease phenotype was assessed as in Fig 3. (**a**) Airway responsiveness (*: P < 0.05 determined by ANOVA), (**b**) bronchoalveolar lavage fluid inflammatory cells, (**c**) total lung IL-4-secreting cells, and (**d**) fungal colony forming units (CFU) recovered from the lungs of infected mice are shown. *: P < 0.05 determined by Kruskal-Wallis test (n = 4 mice/treatment group). Data are from one of 4 independent and comparable biological experiments.

We conducted additional studies to determine the importance of salmeterol-dependent STAT6 activation in vivo. PM-242H appeared to effectively inhibit STAT6 activation in whole lung of IL-13-challenged mice when delivered in a DLPC liposome vehicle intranasally (S9 Fig). Moreover, in a dose-dependent manner, PM-242H almost completely inhibited expression of allergic airway disease in two genetically distinct mouse backgrounds, C57BL/6 (Fig 3) and Balb/c (Fig 4), when given at the initiation of fungal challenge. Thus, PM-242H blocks STAT6 activation in vivo and the initiation of allergic airway disease regardless of mouse genotype.

**Fig 5:**
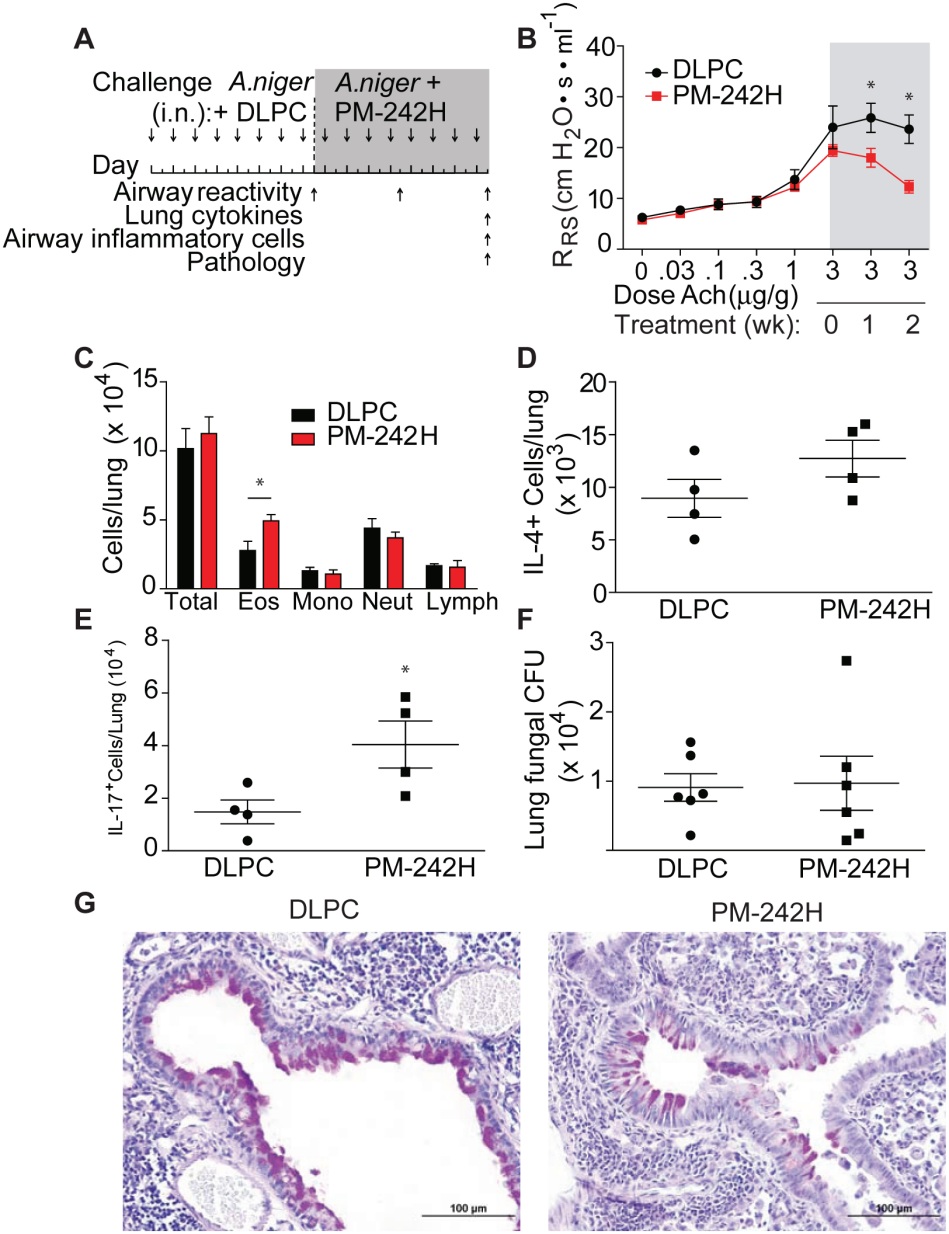
PM-242H reverses established airway hyperresponsiveness. (**a**) Mice were intranasally challenged with *A*. *niger* spores daily i.n. for two weeks after which airway responsiveness to acetylcholine was determined. PM-242H was then given i.n. every other day as spore challenges were continued. Airway responsiveness was determined 7 and 14 days after the initial determination and airway inflammation, lung cytokines and pathology were examined at the end of the experiment. (**b**) Airway responsiveness of vehicle (DLPC) and PM-242H treated animals. *: P < 0.05 determined by ANOVA. (**c**) Total BALF inflammatory cells. (**d, e**) Total lung IL-4 and IL-17A-secreting cells. (**f**) Total recovered lung fungal CFU. (**g**) Representative lung bronchovascular bundles depicting airway epithelial goblet cell metaplasia (periodic acid-Schiff stain; bar represents 100 μm). *: P < 0.05 determined by Mann-Whitney test (n = 4 or 5 mice/treatment group as indicated). Data are from one of 4 independent and comparable biological experiments.

We next determined the ability of PM-242H to reverse previously established allergic airway disease induced with *A*. *niger* spores. Mice with preexisting disease were treated with optimal doses of PM-242H or vehicle while continuing to receive fungal challenges (Fig 5a). All mice developed airway hyperresponsiveness prior to initiation of therapy (Fig 5b). However, mice that subsequently received PM-242H had significantly lower airway hyperresponsiveness compared to vehicle-challenged control animals after one week of therapy and at two weeks showed abrogation of airway hyperresponsiveness (Fig 5b). In contrast, total airway inflammatory cells between control and drug treated mice were similar or higher in PM-242H treated mice and we found similar numbers of IL-4 secreting cells and significantly greater IL-17A- secreting cells from lungs of PM-242H-treated animals (Fig 5c–5e). Despite interrupting a potentially important anti-fungal effector pathway controlled through IL-13 and STAT6 [25], the total number of fungal colonies recovered from the lungs of mice treated with and without PM-242H did not differ (Fig 5f). Histologic analysis for goblet cell metaplasia showed that vehicle challenged mice had significant mucus staining in the airway while PM-242H treated mice had markedly less staining (Fig 5g). Thus, PM-242H inhibited the two major STAT6-dependent components of the allergic airway disease phenotype that underlie airway obstruction, airway hyperresponsiveness and airway goblet cell metaplasia, but did not inhibit previously established allergic inflammatory responses or the ability to control fungal airway growth.

**Fig 6:**
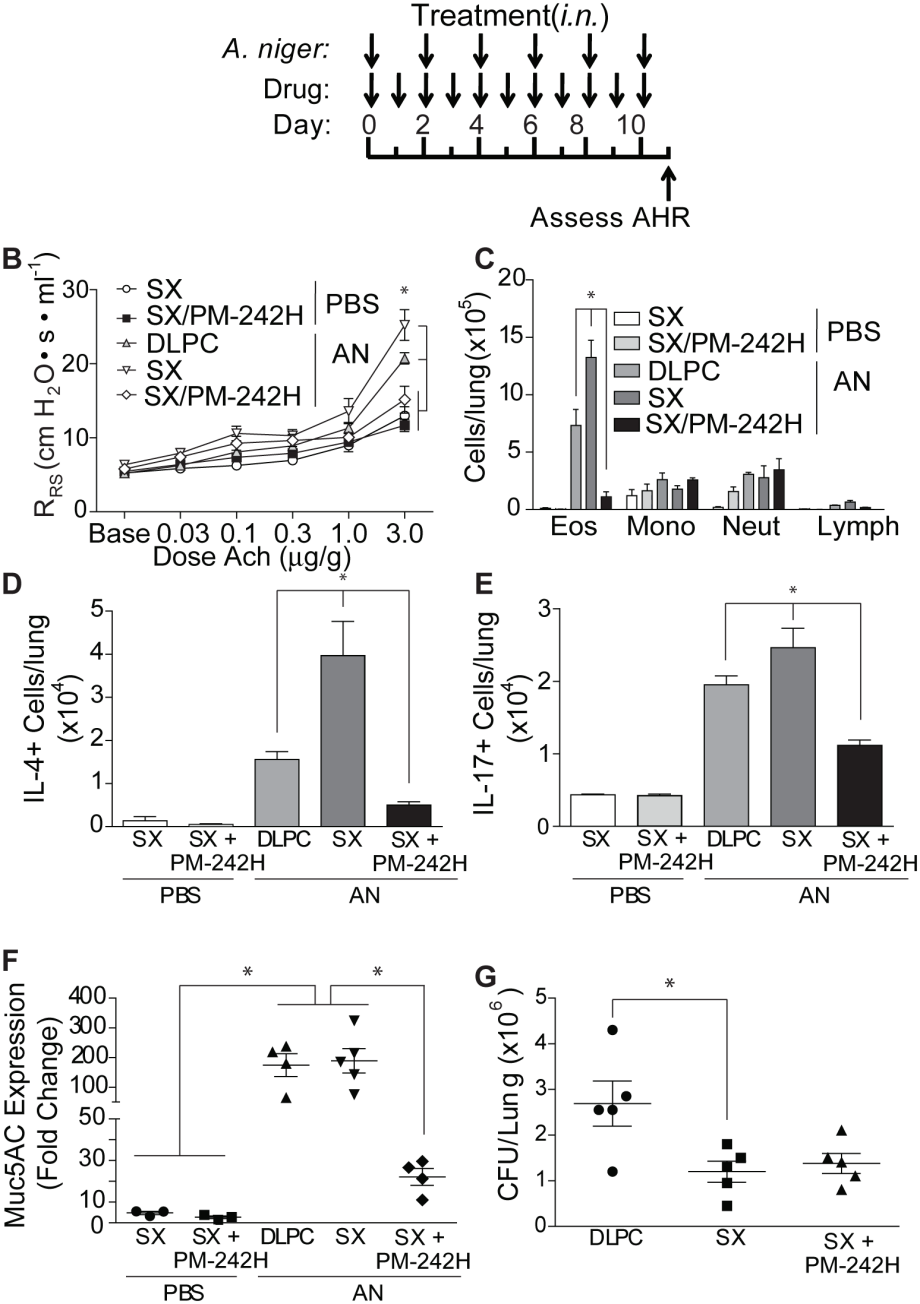
PM-242H reverses salmeterol-dependent exaggerated allergic airway disease. (**a**) Mice were treated daily with DLPC, 50 μg of salmeterol (SX), or salmeterol and PM-242H (50 μg) i.n. and challenged intranasally every other day for 10 days with PBS or *A*. *niger* (AN) i.n. The effects of combined therapy on (**b**) airway hyperresponsiveness (*: P < 0.05 determined by ANOVA), (**c**) total bronchoalveolar lavage fluid (BALF) inflammatory cells (eos: eosinophils; mac: macrophages; lym: lymphocytes; neut: neutrophils), lung (**d**) IL-4- and (**e**) IL-17A-secreting cells, and (**f**) *Muc5AC* expression was determined. (**g**) Total fungal colony forming units (CFU) recovered from lungs of challenged mice. *: P < 0.05 determined by Kruskal-Wallis test (n = 4 or 5 mice/treatment group). Data are from one of 4 independent and comparable biological experiments.

Finally, we determined if STAT6 activation was critical for salmeterol-enhanced disease, testing the effect of PM-242H on salmeterol-enhanced allergic airway disease (Fig 6a). We found that co-administration of PM-242H with salmeterol reversed salmeterol-enhanced disease (Fig 6b). Airway hyperresponsiveness in the combined therapy group was significantly reduced compared to untreated, fungal-challenged mice and was not significantly different from fungus-naïve animals. Lung eosinophilia was also significantly lower in the PM-242H-treated mice compared to both vehicle- and salmeterol- treated mice with allergic airway disease (Fig 6c). Similar trends were observed in total IL-4 (Fig 6d) and IL-17A (Fig 6e) secreting cells from lung homogenates. PM-242H-treated, salmeterol-exposed mice further showed marked reductions in *Muc5ac* mRNA expression (Fig 6f) while still controlling the airway fungal infection (Fig 6g). These studies confirm that the exaggerated allergic airway disease that is induced by salmeterol is mediated through STAT6.

## Discussion

We have shown that structurally related LABAs and other ligands of the β_2_-AR, but not SABAs, enhance allergic airway disease in mice. Moreover, we have shown that structural similarities linking β_2_-AR agonists that enhance allergic airway disease are independent of their classification as agonist, such as salmeterol and formoterol, or as blocker such as carvedilol. Salmeterol enhanced allergic responses through an alternative signaling mechanism involving the β_2_-AR and βarr2 and which involves the suppression of Erk1 and 2 and enhanced activation of STAT6. These findings precisely parallel the epidemiologic link established between LABAs used long-term as monotherapy and loss of asthma control, suggesting that the pro-inflammatory effect of LABAs is due to augmentation of allergic inflammation and T_H_2 responses specifically through activation of STAT6.

Our findings raise important questions regarding the signaling mechanism by which LABAs induce STAT6 activation. Biased agonism through the β_2_-AR is marked over short periods of time in vitro by enhanced Erk1/2 activation [13]. Moreover, in the absence of biased signaling through the β_2_-AR, Erk1/2 activation is also seen in allergic airway disease and Erk suppression attenuates disease expression at least for periods of less than four days of airway allergen challenge [60]. In contrast, although we have shown that βarr2 is required for salmeterol-dependent enhanced airway hyperresponsiveness (Fig 1c), over longer periods of airway allergen challenge (> 4 days), salmeterol signaling causes marked Erk1/2 suppression and STAT6 activation, an effect that is durable for at least two weeks of continuous exposure to β_2_-AR ligand. Thus, our findings suggest that long-term biased agonism through the β_2_-AR with salmeterol and other structurally related ligands may cause Erk1/2 suppression and STAT6 activation, but further studies are required to rule out the existence of another novel signaling mechanism that causes these long-term effects.

Similarly, the strikingly consistent association between β_2_-AR ligands with long aliphatic tails and multiple aromatic groups and enhanced allergic airway disease suggests that these structural motifs are required for the pathogenic activation of STAT6. Crystallographic studies that compare conformations of the β_2_-AR bound to epinephrine and LABAs are needed to extend insights into the effects of ligand structure on receptor confirmation and improve our understanding of the signaling differences, e.g. recruitment of G proteins or βarr2, resulting from these compounds.

Our studies do not refute the ability to LABAs or SABAs to induce short-term broncho-relaxation. Rather, our findings have revealed an additional pro-inflammatory potential of a subset of structurally related LABAs that could exacerbate disease and lead to loss of disease control with long-term, consistent use. As such, our findings provide a molecular explanation of the epidemiologic link noted between long-term use of salmeterol monotherapy and loss of disease control and excess asthma-related mortality [61]. Moreover, as the mechanism has an immune basis involving the aberrant activation of STAT6, our findings further explain the ability of glucocorticosteroids when added to salmeterol to mask in part this untoward effect. Interestingly, addition of glucocorticosteroids to formoterol does not necessarily mask the pro-asthmatic potential, and thus morbidity, of this LABA [15], nor is it clear that addition of glucocorticosteroids entirely block the pro-asthmatic potential of salmeterol with long-term use [14]. Our data further support these observations because although glucocorticosteroids have a clear anti-asthmatic effect (Fig 1C), LABAs activate STAT6 even in the presence of glucocorticosteroids and are alone sufficient to induce asthma-like airway obstruction in the complete absence of allergen provocation (Fig 1D and 1H; S3 Fig).

A limitation of these studies is that they were conducted necessarily in vitro and in vivo using an experimental model of asthma. To make the findings as relevant as possible to humans and human disease, we confirmed major findings in both transformed (A549) and primary human cells (airway epithelial cells, peripheral blood T cells) and further confirmed findings in primary mouse splenocytes. Moreover, the allergic airway disease model has been in use for over 20 years and predicted the successful outcomes of asthma clinical trials involving anti-IL-13 and anti-IL-4Rα antibodies [19, 30, 31]. The data from all of these diverse systems are in agreement and support our hypothesis that LABAs activate STAT6 to promote or exacerbate asthma. Given the inability to perform confirmatory human studies due to their inherent risk, our findings provide a potentially important molecular explanation for the epidemiologic association between use of LABAs and loss of control of asthma and excess asthma-related mortality [11].

We have not examined the allergic disease-promoting properties of all LABAs, but clearly this is a matter of significant current concern and is an issue that should be addressed with any new β_2_-AR ligands destined for clinical use. Fortunately, our findings indicate that the pro-allergic effects of LABAs can, unlike glucocorticosteroids, be fully neutralized by co-administering STAT6 antagonists such as PM-242H, which also hold promise as first-line agents for the treatment of diverse allergic disorders.

## Conclusions

In summary, we have shown that structurally related β_2_-AR agonists such as salmeterol and formoterol and even beta blockers such as carvedilol, but not SABAs such as albuterol, promote exaggerated asthma-like allergic airway disease and enhanced airway constriction in mice. The mechanism underlying this aberrant disease-promoting activity involves the activation of the allergy-dependent transcription factor STAT6 through a βarr2-dependent signaling pathway originating from the β_2_-AR. Our findings provide a molecular explanation for adverse clinical outcomes observed in asthma patients who regularly use LABAs and suggest a template for screening future β_2_-AR drugs for allergic disease-promoting activity. We further introduce a new class of peptidomimetic agent embodied in PM-242H that overcomes the pro-inflammatory potential of LABAs by antagonizing STAT6 activation and which might be therapeutically useful in diverse allergic disease contexts.

## Supporting Information

S1 Fig Structural comparison of common ß_2_-AR drugs. (DOCX)

S2 Fig Scheme for synthesis of compound PM-242H. (DOCX)

S3 Fig Structurally similar ß_2_-AR ligands promote inflammation in lungs. (DOCX)

S4 Fig Salmeterol-enhanced STAT6 activation time course. (DOCX)

S5 Fig Salmeterol-dependent STAT6 activation occurs independently of fluticasone. (DOCX)

S6 Fig Chronic stimulation of human T cells with salmeterol (SX) induces activation of STAT6. (DOCX)

S7 Fig Structure and mechanism of activation of PM-242H. (DOCX)

S8 Fig PM-242H cross-reactivity with STAT transcription factor family members. (DOCX)

S9 Fig Inhibition of Erk1/2 promotes STAT6 activation. (DOCX)

S10 Fig Inhibition of STAT6 activation in vivo. (DOCX)

S1 ARRIVE Checklist ARRIVE Guidelines Checklist. (PDF)
