# Supplementary material for: Long-Acting Beta Agonists Enhance Allergic Airway Disease
Source: PLoS One. 2015 Nov 25;10(11):e0142212. doi: 10.1371/journal.pone.0142212 (PMC4659681; doi:10.1371/journal.pone.0142212)
Supplement: S1 Fig — (DOCX) [file pone.0142212.s001.docx]

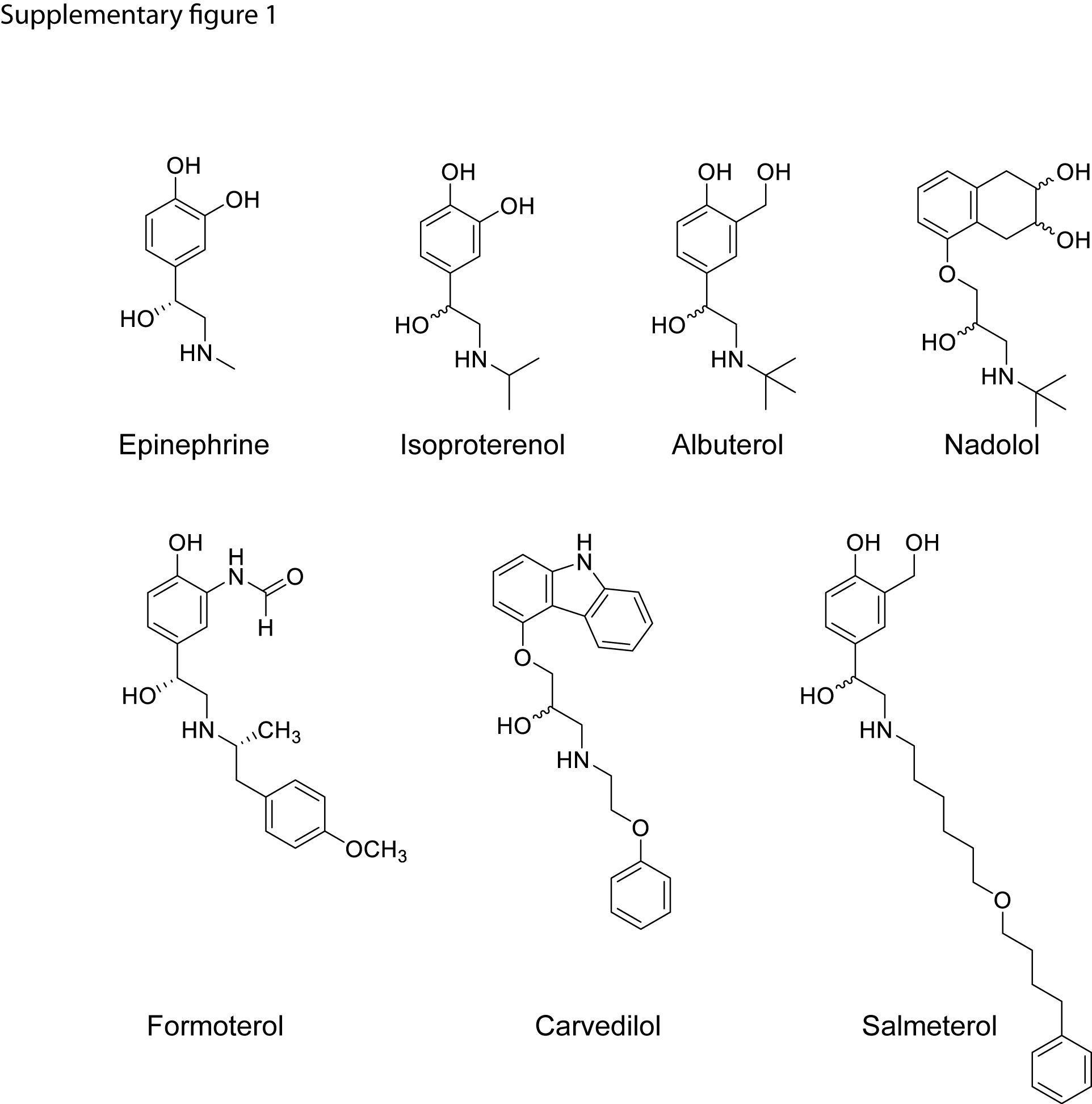


**Figure S1**. Structural comparison of β2-AR drugs. Compared to the natural endogenous ligand epinephrine, the synthetic ligands isoproterenol, albuterol and nadolol all have short aliphatic side chains and only one aromatic group. In contrast, formoterol, carvedilol, and salmeterol all possess longer aliphatic side chains and at least two aromatic groups separated by the aliphatic chain.
