# Supplementary material for: Long-Acting Beta Agonists Enhance Allergic Airway Disease
Source: PLoS One. 2015 Nov 25;10(11):e0142212. doi: 10.1371/journal.pone.0142212 (PMC4659681; doi:10.1371/journal.pone.0142212)
Supplement: S2 Fig — (DOCX) [file pone.0142212.s002.docx]

**Figure S2**. Scheme for synthesis of compound PM-242H. See Materials and Methods for details.
