# Supplementary material for: Long-Acting Beta Agonists Enhance Allergic Airway Disease
Source: PLoS One. 2015 Nov 25;10(11):e0142212. doi: 10.1371/journal.pone.0142212 (PMC4659681; doi:10.1371/journal.pone.0142212)
Supplement: S3 Fig — (DOCX) [file pone.0142212.s003.docx]

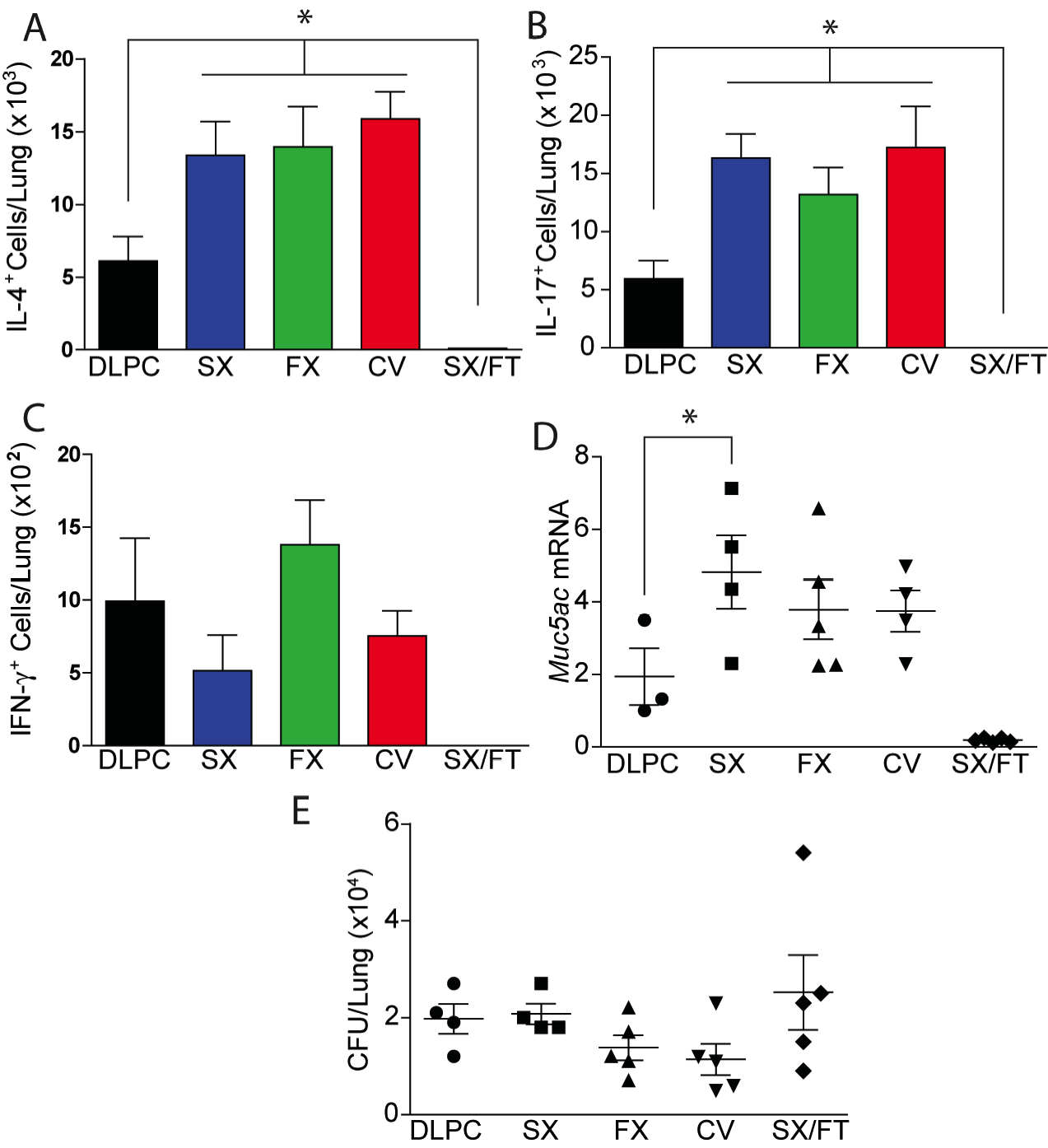


**Figure S3**. Structurally similar β_2_-AR ligands promote inflammation in lungs. Lungs of mice treated i.n. with vehicle (DLPC), salmeterol (SX), formoterol (FX), carvedilol (CV), or salmeterol and fluticasone (SX/FT) and challenged every other day with *A. niger* conidia for 10 days were assessed for IL-4 (**a**), IL-17 (**b**), and IFN-γ (**c**) secreting cells, Muc5ac expression (**d**), and fungal colony forming units (CFU) recovered (**e**). *: P < 0.05 determined by Kruskal-Wallis test (n = 4 or 5 mice/treatment group). Data are from one of 3 independent and comparable biological experiments.
