# Supplementary material for: Long-Acting Beta Agonists Enhance Allergic Airway Disease
Source: PLoS One. 2015 Nov 25;10(11):e0142212. doi: 10.1371/journal.pone.0142212 (PMC4659681; doi:10.1371/journal.pone.0142212)
Supplement: S4 Fig — (DOCX) [file pone.0142212.s004.docx]

**
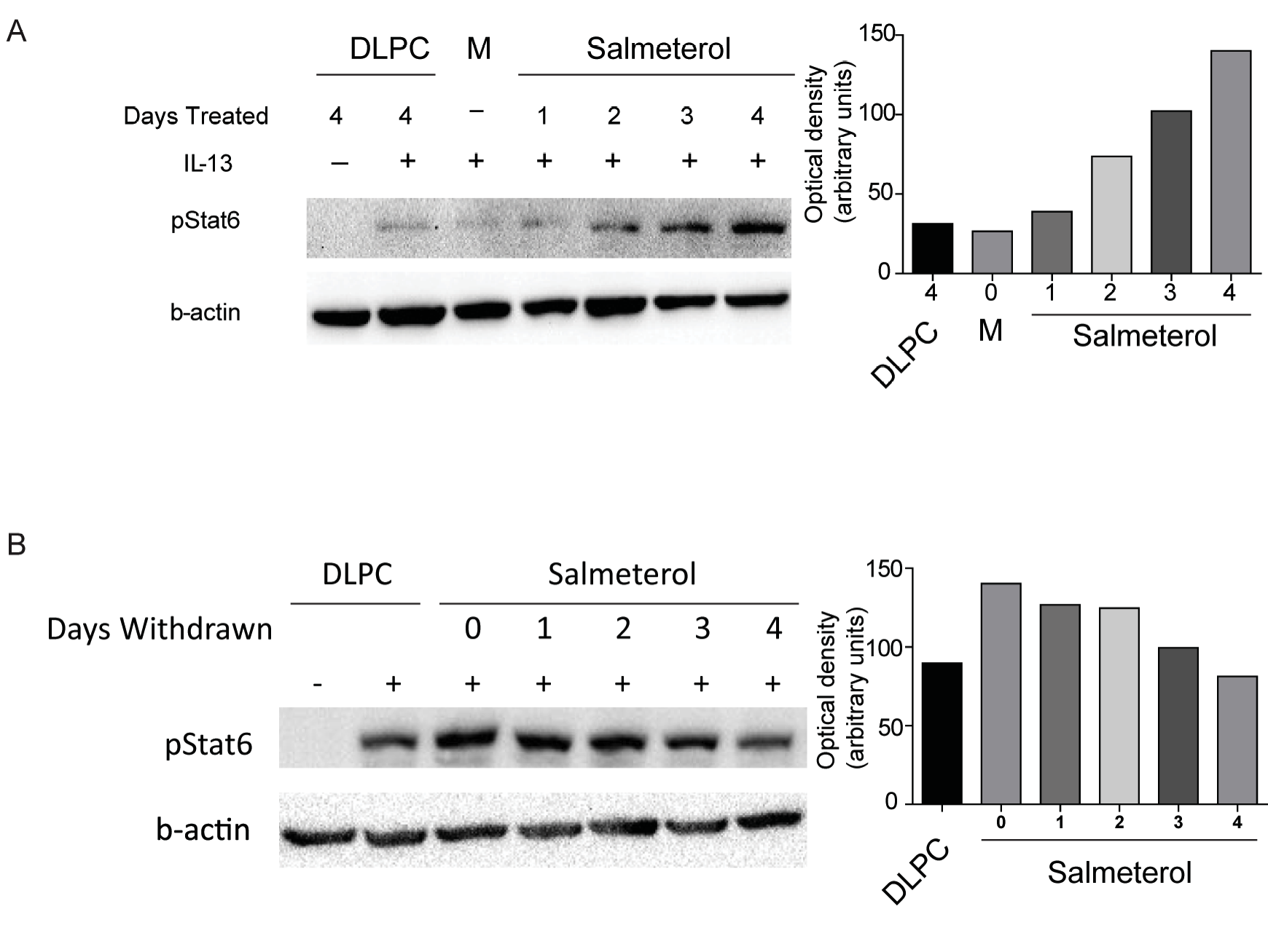
**

**Figure S4**. Salmeterol-enhanced STAT6 activation time course. (A) A549 human airway epithelial cells were cultured in the presence of liposome vehicle (DLPC), media alone (M) or salmeterol for the identified number of days and stimulated with IL-13 for 30 min after which phosphorylated STAT6 was assessed in relation to total beta actin (b-actin). (B) A549 cells were cultures were exposed to DLPC or salmeterol for 5 day after which salmeterol was withdrawn from culture for the indicated number of days and STAT6 phosphorylation was assessed in response to IL-13. Data are from one of 3 independent and comparable biological experiments.
