# Supplementary material for: Long-Acting Beta Agonists Enhance Allergic Airway Disease
Source: PLoS One. 2015 Nov 25;10(11):e0142212. doi: 10.1371/journal.pone.0142212 (PMC4659681; doi:10.1371/journal.pone.0142212)
Supplement: S5 Fig — (DOCX) [file pone.0142212.s005.docx]

**
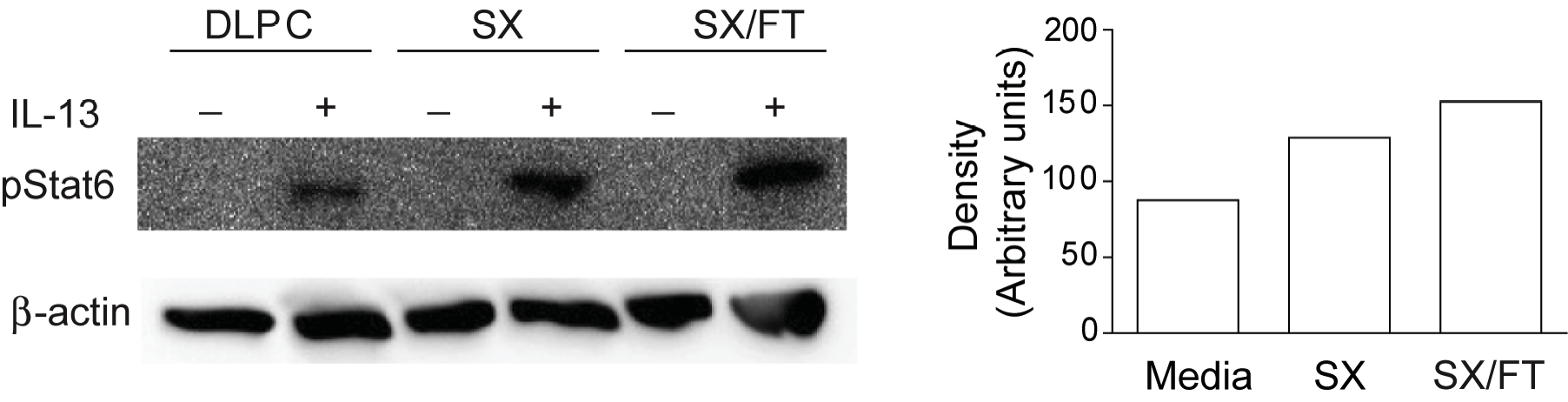
**

**Figure S5**. Salmeterol-dependent STAT6 activation occurs independently of fluticasone. Phosphorylated STAT6 was assessed in A549 cells cultured for 4 days in the presence of liposome vehicle (DLPC), salmeterol (SX), or salmeterol and fluticasone (SX/FT) then stimulated with IL-13 for 30 min. Data are from one 1 experiment.
