# Supplementary material for: Long-Acting Beta Agonists Enhance Allergic Airway Disease
Source: PLoS One. 2015 Nov 25;10(11):e0142212. doi: 10.1371/journal.pone.0142212 (PMC4659681; doi:10.1371/journal.pone.0142212)
Supplement: S6 Fig — (DOCX) [file pone.0142212.s006.docx]

**
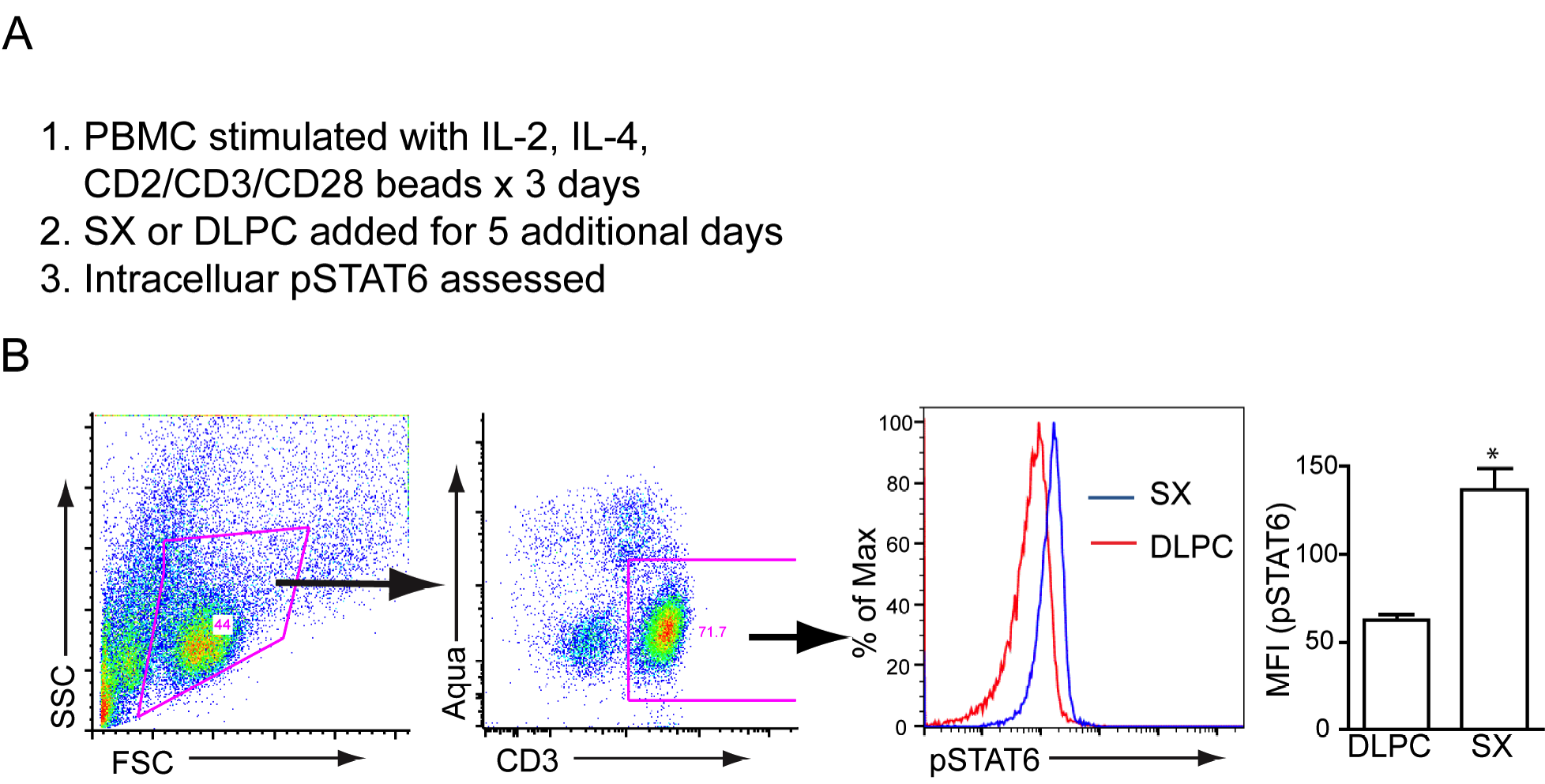
**

**Figure S6**. Chronic stimulation of human T cells with salmeterol (SX) induces activation of STAT6. (A) Human peripheral blood mononuclear cells (PBMC) were initially activated in vitro for 3 days as shown and then stimulated for an additional 5 days with either SX or the vehicle dilauroylphosphatidylcholine (DLPC) after which the cells were permeabilized and a fluorescently conjugated antibody specific for pSTAT6 was added. (B) Representative flow cytometry analysis strategy for intracellular pSTAT6 expression from T cells from a single subject followed by aggregate expression data. Data represent 3 separate experiments involving 3 normal blood donors. *: P < 0.05 relative to DLPC, Mann-Whitney test.
