# Supplementary material for: Long-Acting Beta Agonists Enhance Allergic Airway Disease
Source: PLoS One. 2015 Nov 25;10(11):e0142212. doi: 10.1371/journal.pone.0142212 (PMC4659681; doi:10.1371/journal.pone.0142212)
Supplement: S7 Fig — (DOCX) [file pone.0142212.s007.docx]

**
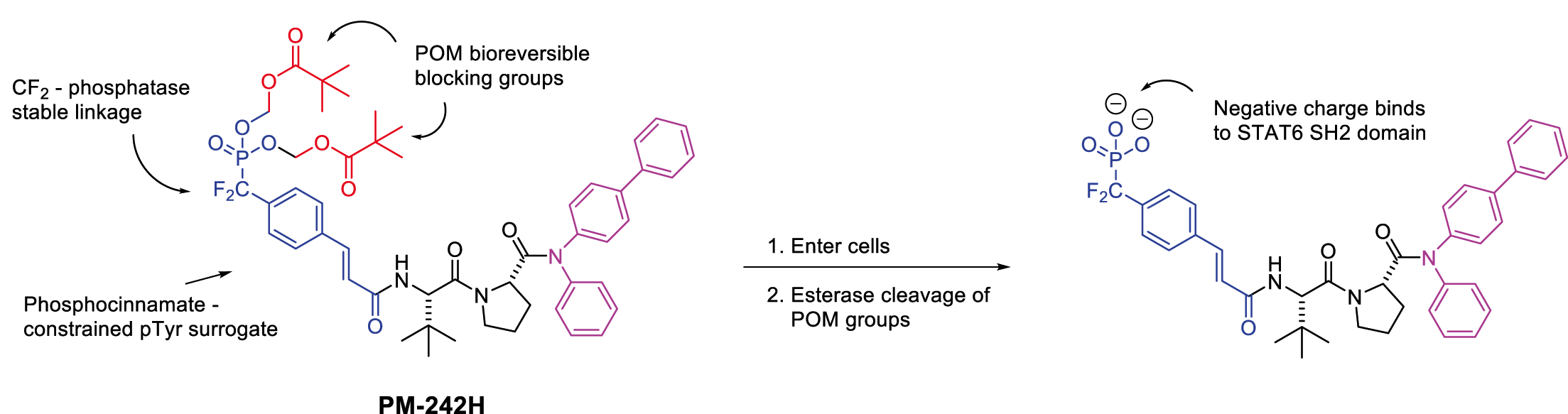
**

**Figure S7**. Structure and mechanism of activation of PM-242H. PM-242 is a phosphatase-stable, cell-permeable prodrug of a phosphopeptide mimic targeting the SH2 domain of STAT6. Phosphotyrosine is replaced by the conformationally constrained surrogate, 4-phosphocinnamic acid (blue). To impart stability to phosphatases, the phosphoryl group is linked to the aromatic ring with a difluoromethyl group. The dipeptide tertiary leucinylproline (black) serves as a scaffold to present benzene rings which impart affinity for the protein (pink). To impart membrane permeability, the phosphosphonate oxygens are blocked with pivaloyloxymethyl (POM) groups (red). On entering cells, carboxyesterase activity cleaves the POM groups resulting in a negatively charged compound, which binds to the phosphopeptide binding area on the surface of the SH2 domain of STAT6. This complex is not able to bind to the pTyr residues on IL-4Rα.
