# Supplementary material for: Long-Acting Beta Agonists Enhance Allergic Airway Disease
Source: PLoS One. 2015 Nov 25;10(11):e0142212. doi: 10.1371/journal.pone.0142212 (PMC4659681; doi:10.1371/journal.pone.0142212)
Supplement: S8 Fig — (DOCX) [file pone.0142212.s008.docx]

**
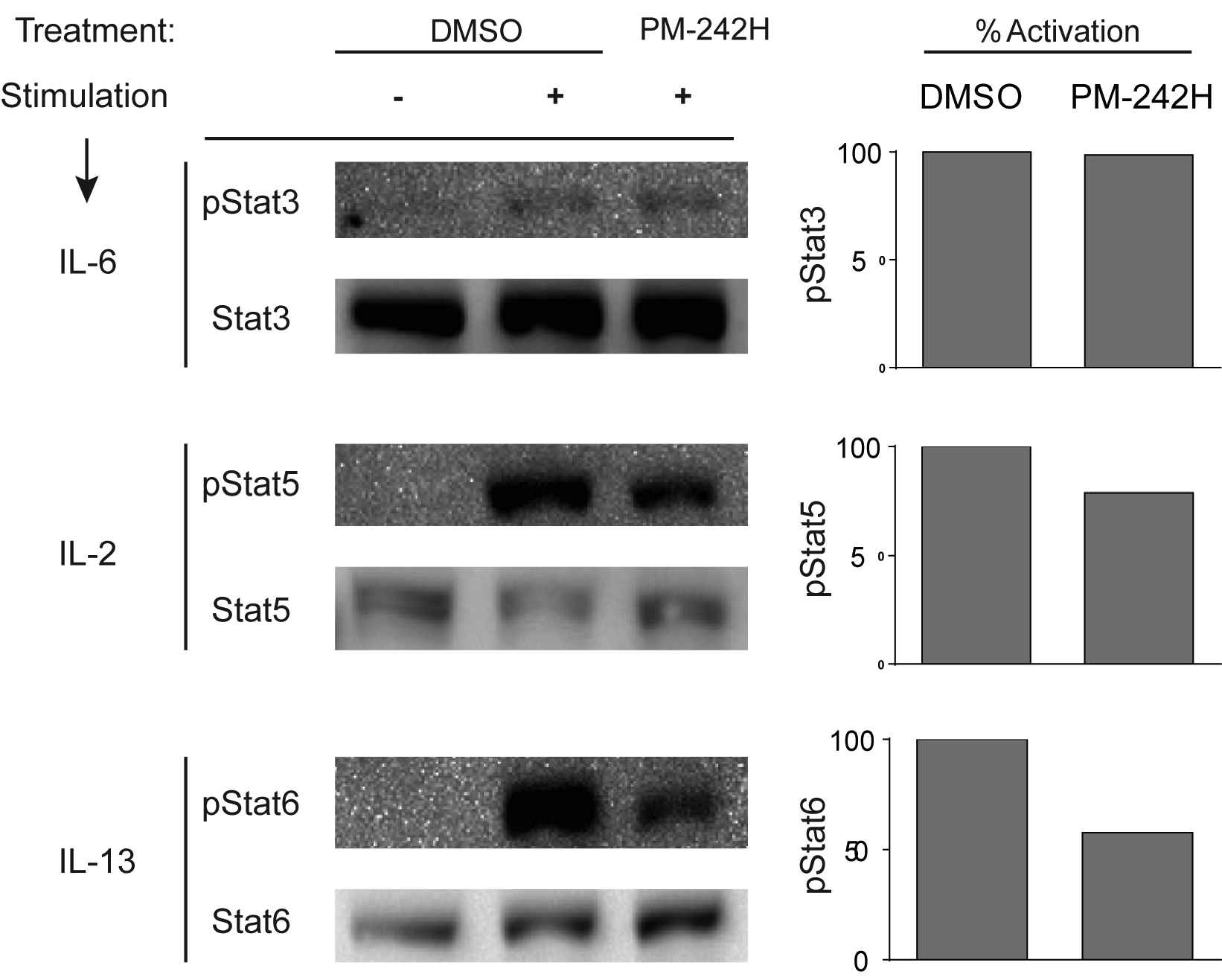
**

**Figure S8**. PM-242H cross-reactivity with STAT transcription factor family members. Murine splenocytes were incubated with vehicle (DMSO) or 242H for 2 hours and stimulated with IL-6, IL-2, or IL-13 for 30 min after which phosphorylation of STAT3, STAT5 and STAT6 was assessed. Data are from one of 3 independent and comparable biological experiments.
