# Supplementary material for: Long-Acting Beta Agonists Enhance Allergic Airway Disease
Source: PLoS One. 2015 Nov 25;10(11):e0142212. doi: 10.1371/journal.pone.0142212 (PMC4659681; doi:10.1371/journal.pone.0142212)
Supplement: S9 Fig — (DOCX) [file pone.0142212.s009.docx]

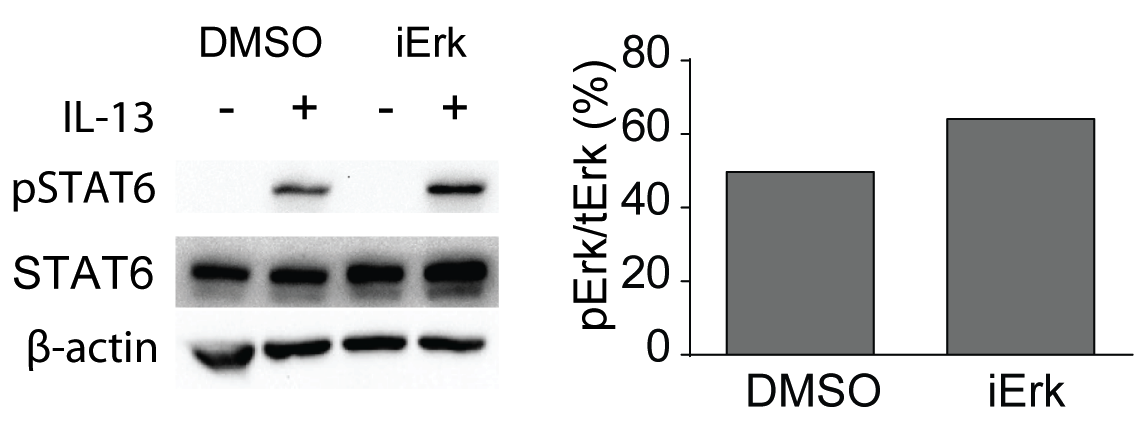


**Figure S9**. Inhibition of Erk1/2 promotes STAT6 activation. Phosphorylated STAT6 was assessed in A549 cells cultured in the presence of vehicle (DMSO) or the Erk1/2 inhibitor U0126 (iErk) for 4 days and stimulated with IL-13 for 30 min. Data are from one of 3 independent and comparable biological experiments.
