# Supplementary material for: Long-Acting Beta Agonists Enhance Allergic Airway Disease
Source: PLoS One. 2015 Nov 25;10(11):e0142212. doi: 10.1371/journal.pone.0142212 (PMC4659681; doi:10.1371/journal.pone.0142212)
Supplement: S10 Fig — (DOCX) [file pone.0142212.s010.docx]

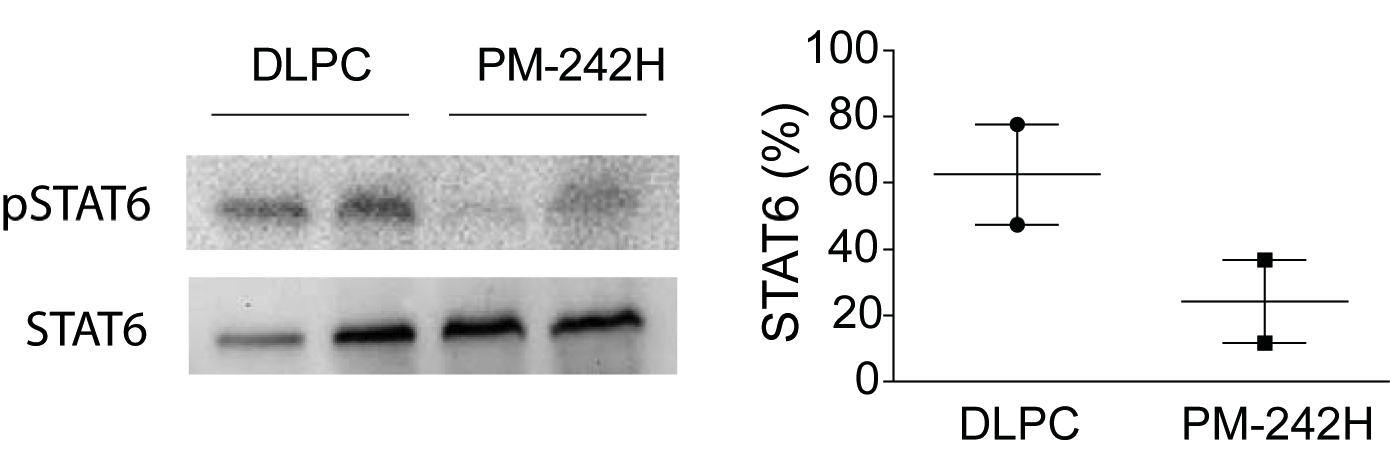


**Figure S10**. Inhibition of STAT6 activation in vivo. Mice (n = 2) were treated (i.n.) with liposome vehicle (DLPC) or 50 μg PM-242H, allowed to rest overnight and challenged (i.n.) with 3.7 μg recombinant IL-13 for 30 min, after which lungs were harvested, perfused with PBS and phosphorylated and total STAT6 were assessed. Data are from one of 2 independent and comparable biological experiments.
